# An Essay on the Philosophy of Medicine

**Published:** 1844-12

**Authors:** 


ART. IV.—*An Essay on the Philosophy of Medicine.* By ELISHA BARTLETT, M.D., Professor of the Theory and Practice of Medicine in the University of Maryland. Philadelphia: Lea & Blanchard. 1844. 8vo. pp. 312.

This is the attractive title of an attractive work, which has just issued from the press of Lea & Blanchard in the best style of that spirited house. It is a work which is likely to excite no little attention, and to provoke not a little animadversion. "The philosophy of medicine,"—how full of euphony the phrase—how promising of interest the discussion of the many topics involved in it! "*I trust*," says the author, quoting John Joachim Becher, "*that I have got hold of my pitcher by the right handle.*" If he has got hold of the true philosophy of medicine, he has made a discovery almost as valuable as that of the philosopher's stone. If he has but indicated clearly the path in which the profession is to advance, and the method by which it is to be purified from the superstitions and follies which have disgraced it for so many centuries, his name will deserve to live in history with that of Bacon. But if we cannot promise him this, nor even assure him that he has got hold of his pitcher by the right handle, we think we can promise him an abundant harvest of criticism. "Whoso taketh up the sword," it has been said, "shall perish by the sword." Our author has grasped the sword with a manly and adroit hand, and has laid about him in good earnest. Not a system-builder has escaped its keen edge. From Cullen to Rush, from Broussais to Thompson, from Hahnemann to Cooke—all are assailed, and he is a lucky man if from each of these schools a swarm of angry combatants is not soon buzzing about his ears. We hope it was not in expectation of such a retaliatory war, that he quoted, in advance, the following passage from Dr. Holmes:

"I know too well the character of these assailants, to gratify their demand for publicity, by throwing a stone into any of their nests. They welcome every cuff of criticism, as a gratuitous advertisement; they grow turgid with delight, upon every eminence of exposure which enables them to climb up where they can be seen. Little as they know of anything, they understand the hydrostatic paradox of controversy; that it raises the meanest disputant to a seeming level with his antagonist: that the calibre of a pipe-stem is as good as that of a water spout, when two columns are balanced against each other. They would be but too happy to figure again in the eyes of that fraction of the public, which knows enough to keep out of fire and water, and to quote that famous line from the idiot's copy-book,

'Who shall decide, when doctors disagree?"'

It must be owned, that while Dr. Bartlett is not sparing of condemnation, he condemns in a courteous and candid spirit. He indulges freely in criticism, and it is piquant and bold; but, at the same time, his instrument seems to be wielded with judgment, and with an eye to the improvement of the profession. His hatred of *rationalism* and his devotion to *numeralism* are, perhaps, excessive; but this is to be said in extenuation of the fault, that his mistress has all the charms of youth to recommend her, and that her rival is old and antiquated. We enter into his feeling. We partake with him in his partialities and antipathies. We are against systems: we are of the *numerical school.*

The author begins with a prefatory chapter on physical science, and lays down and illustrates a series of propositions, the first of which is as follows:

"*Proposition First.*—All physical science consists in ascertained facts, or phenomena, or events; with their relations to other facts, or phenomena, or events; the whole classified, and arranged."

Upon this proposition he makes the following remarks, which, we are sure, are not out of place in such a work. We mean to say, that we are persuaded, with the author, that the truth of this proposition is not properly appreciated. He says:

"The first proposition, that which stands at the head of this chapter, does not require much illustration. Its truth is so manifest, as hardly to admit of any doubt. It would seem almost impossible, that there should be any difference of opinion in reuard to its soundness, or any obscurity in its conception. I believe, nevertheless, it is true, that there has always been, and that there still is, in the minds of most men, and in those of philosophical thinkers, a somewhat imperfect, or confused, apprehension of its doctrines. I do not think that its truth is seen and felt, as it should be, in the simplicity, the purity, and the absoluteness, which belong to it. The confusion to which I allude, is this. There seems to be a common feeling, that the facts, phenomena, and events, with their relationships, classified and arranged, constitute, not the entire science, to which they belong, but only the *foundation* of the science. There is a feeling, that these facts and relations are to be used as elements, out of which, the science is to be built up, or constructed, by what is called *inductive reasoning.* The feeling implies, and the avowed doctrine growing out of it often asserts, that the science *is in this subsequent process of reasoning*, and not in the facts, themselves, and their relationships We are constantly told, that the facts are to be used *as materials*, to be sure; that it is not safe to take for our materials anything but facts; that they constitute the *basis* of every science; but, after all this, the essential condition and constituent of the science is often placed, more in the process of reasoning, as it is called, than in the facts and their relationships. Now, what I wish to insist upon is this; that the science *is in the facts and their relationships, classified and arranged, and in nothing else.* The ascertained facts and their relationships, classified and arranged, constitute, in themselves, and alone, the science, and the whole science, to which they belong.

Prefatory to the second part of his work, our author lays down the following propositions:

*Proposition First.*—All medical science consists in ascertained facts, or phenomena, or events; with their relations to other facts, or phenomena, or events; the whole classified and arranged.

"*Proposition Second.*—Each separate class of facts, phenomena, and events, with their relationships, constituting, as far as they go, medical science, can be ascertained in only one way; and that is by observation, or experience. They cannot be deduced, or inferred, from any other class of facts, phenomena, events, or relationships, by any process of induction, or reasoning, independent of observation.

"*Proposition Third.*—An absolute law, or principle, of medical science consists in an absolute and rigorous generalization of some of the facts, phenomena, events, or relationships, by the sum of which the science is constituted. The actual, ascertainable laws, or principles, of medical science are, for the most part, not absolute but approximative.

"*Proposition Fourth.*—Medical doctrines, as they are called, are, in most instances, hypothetical explanations, or interpretations, merely, of the ascertained phenomena, and their relationships, of medical science. These explanations, consist of certain other assumed and unascertained phenomena and relationships. They do not constitute a legitimate element of medical science. All medical science is absolutely independent of these explanations.

"*Proposition Fifth.*—Diseases, like all other objects of natural history, are susceptible of classification and arrangement. This classification and arrangement will be natural and perfect just in proportion to the number, the importance, and the degree of the similarities and the dissimilarities between the diseases themselves."

He then goes on to add the following remarks, which we give in nearly his own language. Our knowledge of anatomy is not dependent upon our knowledge of other branches of medical science. Our knowledge of physiology is not deducible from our knowledge of anatomy. Our knowledge of pathology is not deducible from our knowledge of pathology. Our knowledge of the causes of disease is the exclusive result of observation; consequently etiology is not to be deduced from pathology. Finally, our therapeutics are not deducible from our pathology. In amplification of the latter proposition, he adds as follows:

"Writers upon the science and the art of medicine have always been, so far as the subject now before us is concerned, divided into two classes, or schools; those of the *rationalists*, and of the *empirics*. The former have always been, and still continue to be, the most numerous and powerful. Their doctrines have pervaded and governed the medical world. They claim to be more *philosophical*, than their opponents, the empirics. They profess to be governed and guided, in their theory and practice, by what they are pleased to call *rational principles*. They allege, tbat their therapeutics is founded upon rational *indications*. They claim, not merely to cure diseases, but to cure them *philosophically*, and in conformity to their *rational principles*. They claim, not merely to have ascertained the relationship, which exists between diseases and their remedies, but to understand the *nature* and the *reasons* of this relationship. They pretend to explain the *mode* and *manner* in which these remedies produce their results. Their doctrine is, that therapeutics is *founded upon* pathology; that the former is deduced from the latter. They are very confident in their knowledge of the intimate *modus*

*operandi* of their remedies. The empirics, on the other hand, deny all this. They say, that the whole science and art of therapeutics are founded upon simple experience. They say, that our knowledge of the relationship between disease and the modifiers, is the sole and exclusive result of observation of this relationship itself. They disclaim any knowledge of the intimate and essential naturé of this relationship. They deny, that any acquaintance, however complete and accurate, with the phenomena of pathology, could ever, of itself, have led to a knowledge of the relations, which exist between these phenomena, and those substances and influences in nature, endowed with the property of arresting or controlling these phenomena. They deny, that therapeutics is founded upon pathology. They deny, that by any process of reasoning, the former can be deduced from the latter. This doctrine, I hardly need say, is the doctrine of this essay; and the remaining portion of the present chapter will be devoted to its statement and illustration."

The following questions which he puts, in illustration of his views, it will be difficult to answer:

"An examination of all the other therapeutical relationships of inflammation will render the principle, which I am endeavoring to illustrate, still clearer and more evident. There is not one amongst them, which could have been indicated, even, by any methoa of deductive reasoning. How could any such reasoning have ever led to the conclusion, that the abstraction of blood from the general circulation would have diminished the intensity, or shortened the duration, or in any way changed the action of this local morbid process? How could any such reasoning have led to a knowledge of the circumstances, in which this abstraction of blood would be followed by beneficial results? How could any such reasoning have led to a knowledge of the relationships, which exists between inflammation, and the operation of calomel, antimony, and opium? Could the effects of these substances have been deduced, or inferred, from any knowledge, however accurate, of the phenomena of inflammation? Manifestly, and indisputably, not. All these effects have been ascertained by simple and direct observation of the effects themselves. It is not possible, in the nature of things, that they could have been ascertained by any other method, or in any other way.

"There are certain pathological processes and conditions, one characteristic element of which consists in a distinct and well marked periodicity in their recurrence. These processes and conditions differ very widely from each other in many important particulars; but they agree in this. The most common of these are intermittent fever, and periodical neuralgia. Perhaps there is no therapeutical relationship better established, than that which exists between these diseases, on the one hand, and cinchona and arsenic, on the other. These substances, when introduced into the system, are endowed with the power of arresting, or of modifying, the above-mentioned diseases. Is there anything in this periodical element of these diseases, which, by any process of deduction, could have led to a knowledge of its relationship to these substances? Do these substances possess any other known property in common, excepting this of their relationship to these diseases? There can be but one answer to all these questions. No attainable knowledge of the morbid element; no attainable knowledge of these substances, could have ever led, independent of experience, to a knowledge of the relation, which exists between them. Who could have anticipated, that the action of an emetic would relieve the difficult breathing of croup? What *rational* connexion is there between syphilis and the preparations of mercury; or between scrofula and iodine?"

Thus, we have given enough of this work to exhibit the scope and bearing of its doctrines, and the purity and elegance of the style in which it is written, and that is all we proposed at present. When we shall enjoy more leisure than is now at our command, we may return to it for the purpose of examining some of its opinions in which we do not concur; but in the meantime we commend it to our readers as a work of thought, of labor, and of polish—a work betokening a mind at once logical and poetical—a mind possessing a keen relish for the beauties of nature, but capable of engaging in the drudgeries of practical life. Y.

